# Dialogue or Mechanism Versus Vitalism

**Published:** 1840

**Authors:** 


### Dialogue between a Physician and a Physiologist; or Mechanism *versus* Vitalism.

[*Scene a Laboratory.*]

*Physician.*—Is it true, my dear Magendie, as reported in your lectures, that you can produce *inflammation* in the dead body?

*Magendie.*—I can.

*Physician.*—The cardinal points or phenomena of inflammation, as laid down by all writers, from Celsus to the present time, are rubor, tumor, calor, dolor.

*Megendie.*—Very well.

*Physician.*—Can you produce *rubor* in the dead tissues, and if so, by what means?

*Megendie.*—Nothing more easy. I dip the parts in red ink, or inject the *vessels* with red wax.

*Physician.*—The tumor?

*Magendie.*—By injecting the *cellular* membrane with warm fluids of any kind.

*Physician.*—The calor?

*Magendie.*—I immerse the parts in hot water, or hang them before the fire.

*Physician.*—Humph! These are very mechanical processes for producing the first three phenomena of inflammation.

*Magendie.*—Doubtless. But all Nature's processes, in health or disease, are mechanical, quite as much so those by which I imitate her operations.

*Physician.*—There is one phenomenon more, however, which I think will pose you. How do you produce the dolor?

*Magendie.*—There you are in the clouds and fogs of vitalism—clouds and fogs by which you strive to conceal your ignorance. Sir I admit the existence of no phenomena or process in the living or in the dead body, but what can be made cognizable to the senses. We have no other inlets of knowl- than through the five senses. Now, sir, I ask you, can you *see* pain? No. Can you *touch* pain? No. Can you *hear* pain? No. Can you *smell* pain? No. Can you *taste* pain? No. Then, sir, what monstrous absurdity is it to talk of a phenomenon presenting itself, you say, in a patient before you, and of which you cannot learn the most minute iota by the evidence of your own senses! Sir, I repudiate, scorn—nay, detest, all those phenomena and explanations which are founded on vitalism—and thus I throw them to the winds. While pronouncing these last words, with great violence of gesture, and action of his arms, which he waved over his head, he struck his right hand with such force against a lamp which hung in the laboratory, that he roared with agony, and thrust his knuckles into his mouth to mitigate the *pain!*

*Physician.*—Ah! friend Magendie! what say you to the evidence of the senses now? Here M. Magendie flounced out of the laboratory, and slammed the door in the physician's face, who was following him in a convulson of laughter.—*Ib.*

